# Association of tobacco use with depressive symptoms in adults: Considerations of symptom severity, symptom clusters, and sex

**DOI:** 10.1371/journal.pone.0319070

**Published:** 2025-04-02

**Authors:** Shakila Meshkat, Qiaowei Lin, Vanessa K. Tassone, Reinhard Janssen-Aguilar, Hilary Pang, Wendy Lou, Venkat Bhat

**Affiliations:** 1 Interventional Psychiatry Program, St. Michael’s Hospital, Toronto, Ontario, Canada; 2 Department of Biostatistics, Dalla Lana School of Public Health, University of Toronto, Toronto, Ontario, Canada; 3 Institute of Medical Science, Temerty Faculty of Medicine, University of Toronto, Toronto, Ontario, Canada; 4 Department of Psychiatry, University of Toronto, Toronto, Ontario, Canada; 5 Neuroscience Research Program, St. Michael’s Hospital, Toronto, Ontario, Canada; University of Botswana School of Medicine, BOTSWANA

## Abstract

**Objective:**

We aim to assess the association between depressive symptoms, depressive symptom severity and symptom clusters with tobacco use. We will also evaluate sex differences in these associations.

**Method:**

This cross-sectional study used data from the National Health and Nutrition Examination Survey (2005-2018). Depressive symptoms were assessed using the Patient Health Questionnaire-9. Tobacco use was categorized into four groups: cigarette use, smoked tobacco products (pipes and cigars), smokeless tobacco products (chewing tobacco and snuff), and non-tobacco use (reference group).

**Results:**

This study included 33,509 participants. Cigarette use was associated with a 0.83-unit increase in total PHQ-9 scores (95% CI =  [0.63, 1.04]), and 1.73 times higher odds of reporting depressive symptoms (95% CI =  [1.48, 2.02]) compared to non-tobacco use. However, the use of smoked and smokeless tobacco was not associated with depressive symptoms. In females, cigarette use showed a stronger association with total PHQ-9 scores (aCoef =  1.23, 95% CI =  [0.92, 1.55]) than in males (aCoef =  0.45, 95% CI =  [0.21, 0.69]). Additionally, female smoked tobacco users showed positive associations with both PHQ-9 scores and the presence of depressive symptoms, but this relationship was not observed in males. Furthermore, subgroup analysis revealed associations between cigarette use and cognitive-affective and somatic symptom clusters, as well as a relationship between the logarithm of total cigarette consumption and depressive symptoms.

**Conclusion:**

Cigarette use was associated with higher odds of depressive symptoms with females having a stronger association. Further studies are needed to replicate these findings and examine the underlying mechanisms.

## 1. Introduction

Major depressive disorder (MDD) is a common and disabling mood disorder that affects 5% of adults worldwide [1]. The prevalence of depression in the United States (US) is estimated to be 21% in females and 10% in males [2,3]. Depression is conceptualized as a combination of two symptom clusters—somatic and cognitive-affective—rather than a singular, uniform condition [4]. In accordance with the Diagnostic and Statistical Manual of Mental Disorders fifth edition MDD criteria, somatic symptoms encompass facets such as sleep, energy, appetite, and psychomotor slowing or restlessness [4,5]. Concurrently, the cognitive-affective cluster includes anhedonia, low mood, guilt, concentration difficulties, and suicidal ideation [5]. Importantly, the cognitive-affective cluster is associated with an individual's perception of unmet psychological care needs, distinguishing it from somatic symptoms [5]. Research suggests that analyzing these symptom clusters provides more insightful perspectives than exclusively focusing on overall depression, particularly concerning health outcomes [6].

Tobacco use remains a significant global public health issue, with nearly one billion smokers worldwide, contributing to millions of deaths annually [7,8]. A growing body of evidence links tobacco use to psychiatric illnesses, including depression, though the relationship remains debated [9,10]. Several studies have demonstrated a consistent observational association between smoking and depression. For instance, a cross-sectional analysis using World Health Organization/International Society on Biomedical Research on Alcoholism study data indicated a positive association between depression and smoking [11]. Further, a study by Wootton et al. on 462,690 participants from the UK Biobank utilized Mendelian randomization to estimate the causal effect of lifetime smoking on the risk of MDD, highlighting an increased risk for both lifelong and new smokers [12]. However, conflicting findings exist. For example, Munafò et al. reported no significant association between depression and smoking status using data from National Longitudinal Study of Adolescent Health [13]. It remains unclear whether the smoking-depression relationship is causal or driven by a complex interplay of biological, psychological, social, and cultural factors. Moreover, there is a need for further exploration of sex differences in the association between depression and smoking, as previous studies have yielded inconsistent results.

Furthermore, existing research often focuses solely on cigarette smoking, with limited attention to other tobacco products or symptom clusters related to depression [10,14]. Thus, evaluating sex differences and different symptom clusters in tobacco use remains crucial. This study aims to expand on previous research, such as Fan et al. (2022) [15], which used NHANES data to examine sex differences in the association between tobacco smoking and depressive symptoms. We seek to improve on prior work by further exploring how depressive symptoms, their severity, and symptom clusters relate to tobacco use, with a particular focus on sex differences. We hope to contribute new insights into the nuanced relationship between depression and tobacco use across various populations.

## 2. Methods

### 2.1. Study population

Data for this study were sourced from the 2005 to 2018 National Health and Nutrition Examination Survey (NHANES). NHANES is an annual survey conducted by the National Center for Health Statistics (NCHS), a branch of the Centers for Disease Control and Prevention (CDC). It aims to assess the health and nutritional status of the non-institutionalized civilian population in the US. This survey is unique due to its combination of interviews and physical examinations. Ethical approval for NHANES data collection protocols, including securing written informed consent from all participants, is provided by the NCHS Research Ethics Review Board. The NHANES database encompasses a wide range of demographic, socioeconomic, dietary, and health-related information, making it a valuable resource for epidemiological studies and public health research. Detailed descriptions of the survey methodologies, including the sampling procedures and data collection methods, are available on the CDC website (https://wwwn.cdc.gov/nchs/nhanes/analyticguidelines.aspx#). The study population was restricted to females and males aged 20 years and older who had completed both the Mental Health - Depression Screener (DPQ) and the Smoking - Recent Tobacco Use (SMQRTU) questionnaires.

### 2.2. Exposure variable

The main analysis focused on various forms of tobacco use, including cigarettes, smoked tobacco products, smokeless tobacco products, and non-tobacco use. The categorization of tobacco use was based on responses to multiple-choice questions from the SMQRTU questionnaire, which inquired about the use of different tobacco products within the last five days (SMQ690A/B/C/D/E). Participants could choose from cigarettes (SMQ710), pipes (SMQ740), cigars (SMQ770), chewing tobacco (SMQ800), and snuff (SMQ817), and were further asked to specify the number of days they used each respective product within the last five days. ‘Cigarette’ users were those who responded with a “1” on SMQ690A or any value from 1 to 5 on SMQ710, and reported no use of other tobacco products. Similarly, ‘Smoked Tobacco Products’ was determined by positive responses to the pipes and cigars questions (SMQ690B/C) or the corresponding frequency questions (SMQ740, SMQ770), with the term referring exclusively to products other than cigarettes. ‘Smokeless Tobacco Products’ was determined by responses to the chewing tobacco and snuff related questions (SMQ690D/E, SMQ800, SMQ817). ‘Non-Tobacco Use’ was defined as individuals who answered “No” to questions SMQ680 or SMDANY, which asked if they had “used any tobacco product in the last 5 days?.”

Sub-analysis 1 focused on the comparison between the ‘Cigarettes’ and ‘Non-Tobacco Use’ groups for the tobacco use variable. In sub-analysis 2, total cigarette consumption was calculated by multiplying the number of days smoked (SMQ710) by the number of cigarettes smoked per day (SMQ720), reflecting the total cigarette use over the past five days. This measure was designed to quantify exposure for an in-depth analysis of the association between cigarette use and depressive symptoms.

### 2.3. Outcome variable

Depressive symptoms were evaluated using the nine-item Patient Health Questionnaire (PHQ-9), which measures the frequency of depressive symptoms within the last two weeks [16]. The PHQ-9 queries are scaled to reflect the severity of symptoms: 0 (“not at all”), 1 (“several days”), 2 (“more than half the days”), and 3 (“nearly every day”). The overall severity of depressive symptoms is measured by the sum of all item scores, providing a range from 0 to 27. A score of 10 or higher indicates clinically meaningful depressive symptoms, whereas a score below 10 suggests no depressive symptoms. Additionally, depressive symptom severity was categorized into five levels: minimal (0-4), mild (5-9), moderate (10-14), moderately severe (15-19), and severe (20-27). Several studies have demonstrated the validity and reliability of the PHQ-9 for depression [16,17].

The PHQ-9 is also indicative of cognitive-affective and somatic depressive symptoms. The cognitive-affective score was computed by totaling the scores from questions 1, 2, 6, 7, and 9, while the somatic score was the sum of the responses to questions 3, 4, 5, and 8. This method of segmentation allows for an in-depth examination of depressive symptom clusters and their potential correlations with tobacco use patterns. In sub-analysis 1, the study examined cognitive-affective and somatic depressive symptoms as continuous variables.

### 2.4. Covariates

Covariates included sex (female or male), age (1 =  [20-39], 2 =  [40-59], 3 =  [60-79], 4 =  [>=80]), race (Mexican American, other Hispanic, non-Hispanic White, non-Hispanic Black, or other race including multi-racial), education (less than 9th grade, 9th-11th grade, high school graduate/General Educational Development (GED), some college/Associate of Arts (AA) degree, and college graduate or above), marital status (never married, married, widowed, divorced, separated, or living with a partner), ratio of family income to poverty (PIR; low income < 1, middle income 1 to 4, and high income >  4) [18], body mass index (BMI; underweight <  18.5 kg/m^2^, healthy weight =  18.5 to 24.9 kg/m^2^, overweight =  25 to 29.9 kg/m^2^, obese ≥  30 kg/m^2^), sleep duration at night on weekdays or workdays (short (<7 hours), average (7–9 hours), and long (>9 hours) [19], substance use (yes to ever used marijuana or hashish, or ever used cocaine/heroin/methamphetamine, or ever used a needle to inject illegal drugs; no to all of the above) [20], and history of health indicators such as diabetes, hypertension, heart failure, angina, stroke, and cancer [21]. The presence of these diseases was treated as a binary variable, with ‘Yes’ indicating the presence and ‘No’ indicating the absence of the condition.

### 2.5. Statistical analysis

#### 2.5.1. The analyses conducted in this study were not pre-registered.

R studio (version 4.4.0) was used for statistical analyses, with the ‘survey’ package to incorporate survey weights. To adjust for the combination of seven survey cycles, Mobile Examination Center (MEC) survey weights were divided by a factor of seven. In the demographic characteristics table, categorical variables were presented as unweighted frequency and weighted percentages, while continuous variables were reported using weighted mean along with the standard deviation (SD). A table providing a quantitative overview of tobacco product usage was generated based on responses to questions regarding the ‘number of days smoked (specific tobacco type) in the last 5 days’ (SMQ710 - SMQ817), treating each tobacco type as mutually exclusive. Linear regression analysis examined the association between tobacco use and PHQ-9 scores. Logistic regression models were applied to explore the association between tobacco use and the presence of depressive symptoms. Multiple logistic regression was conducted to evaluate the relationship between tobacco use and the severity of depressive symptoms, stratifying by sex when a significant interaction between sex and tobacco use was observed.

In the sub-analysis 1, a symptom profile distribution map was created to compare the nine individual depressive symptoms between cigarette users and non-tobacco users. The proportions for each response level (i.e., 0-3) for each item of the DPQ (DPQ010 - DPQ090) were calculated within the group of cigarette users and non-tobacco users by dividing the number of individuals who selected a particular response (e.g., 0) by the total number of individuals who answered the question. The association between tobacco use (cigarettes vs. non-tobacco use) and scores of cognitive-affective and somatic symptom clusters was determined using linear regression models. Additional models included interaction terms between tobacco use and sex. When the interaction effects were statistically significant, subgroup analyses by sex were conducted. Sub-analysis 2 replicated the analytical approach of the main analysis, accommodating various forms of depressive symptoms (continuous, binary, or ordinal). A logarithmic transformation was applied to the total cigarette exposure variable to meet model assumptions.

Covariates were selected based on clinical relevance, informed by prior knowledge, team expertise, and findings from the literature. References have been added at the end of each covariate in the covariates section. All adjusted models consistently included the selected covariates. We opted not to perform imputation for the missing survey data to avoid introducing potential bias or inaccuracies. Thus, listwise deletion was utilized to handle missing data, which involves removing any cases from the analysis that have missing values in any of the variables included in the model.

## 3. Results

### 3.1. Descriptive statistics

**Fig 1:**
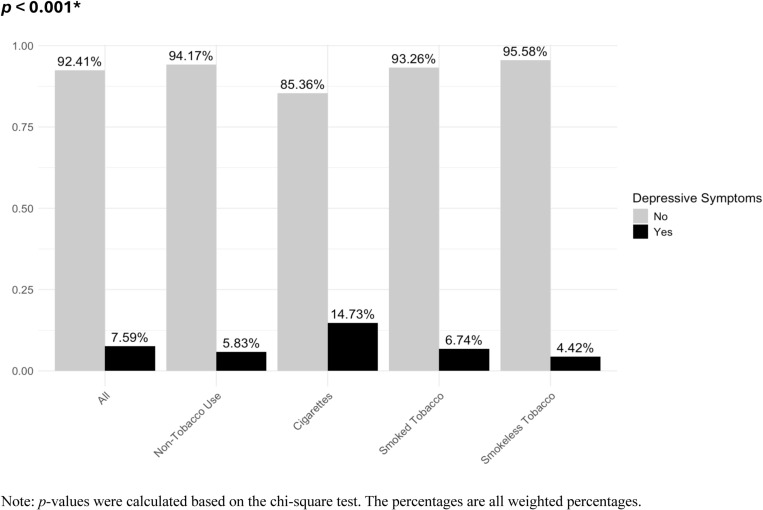
Prevalence of depressive symptoms by tobacco use category.

**Fig 2:**
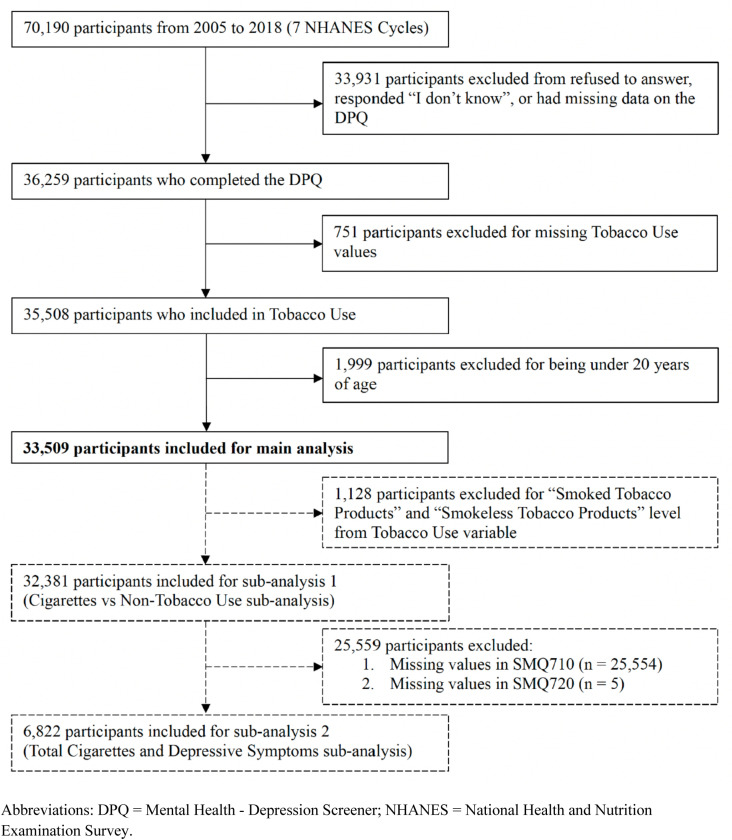
Participant inclusion flowchart.

The study population involved 33,509 participants, including 17,223(51.81%) females. Among these, 2,917participants (7.59%) had depressive symptoms (Fig 1). Moreover, 25,553 participants (76.17%) were non-tobacco users, 6,828 (19.92%) were cigarette users, 633 (1.85%) used smoked tobacco products, and 495 (2.06%) used smokeless tobacco products (Table 1). Fig 2 illustrates the inclusion of participants in the study. Table 1 summarizes all variables involved in the main analysis, stratified by the presence of depressive symptoms.

**Table 1:** Demographic characteristics, stratified by presence of depressive symptoms (n =  33,509).

Characteristic	Total	Depressive Symptoms No	Depressive Symptoms Yes	*p*-value
Sample Size	33509	30592	2917	–
Total PHQ-9 Scores (mean (SD))	3.00 (4.04)	2.09 (2.37)	14.04 (3.79)	< 0.001
Depressive Symptom Severity (%)				< 0.001
Minimal	25330 (77.25)	25330 (83.59)	0.00 (0.00)	
Mild	5262 (15.16)	5262 (16.41)	0.00 (0.00)	
Moderate	1818 (4.84)	0.00 (0.00)	1818 (63.76)	
Moderately Severe	788 (1.98)	0.00 (0.00)	788 (26.14)	
Severe	311 (0.77)	0.00 (0.00)	311 (10.10)	
Tobacco Use (%)				< 0.001
Non-Tobacco Use	25553 (76.17)	23802 (77.62)	1751 (58.52)	
Cigarettes	6828 (19.92)	5760 (18.38)	1068 (38.64)	
Smoked Tobacco Products	633 (1.85)	568 (1.86)	65 (1.64)	
Smokeless Tobacco Products	495 (2.06)	462 (2.13)	33 (1.20)	
Sex (%)				< 0.001
Male	16286 (48.19)	15239 (49.26)	1047 (35.18)	
Female	17223 (51.81)	15353 (50.74)	1870 (64.82)	
Age (%)				< 0.001
20 – 39	11118 (35.91)	10233 (36.02)	885 (34.61)	
40 – 59	10898 (37.47)	9757 (37.04)	1141 (42.65)	
60 – 79	9430 (22.39)	8655 (22.61)	775 (19.79)	
>= 80	2063 (4.23)	1947 (4.33)	116 (2.95)	
Race (%)				< 0.001
Mexican American	5307 (8.48)	4851 (8.50)	456 (8.23)	
Other Hispanic	3201 (5.47)	2824 (5.29)	377 (7.66)	
Non-Hispanic White	14288 (67.77)	13063 (68.13)	1225 (63.37)	
Non-Hispanic Black	7169 (10.98)	6525 (10.77)	644 (13.51)	
Other Race - Including Multi-Racial	3544 (7.30)	3329 (7.31)	215 (7.23)	
Education (%)				< 0.001
Less than 9th grade	3411 (5.12)	2977 (4.84)	434 (8.51)	
9-11th grade	4716 (10.37)	4101 (9.82)	615 (17.01)	
High school graduate/ GED	7722 (23.30)	7015 (22.98)	707 (27.18)	
Some college/ AA degree	9904 (31.51)	9044 (31.35)	860 (33.47)	
College graduate or above	7730 (29.70)	7432 (31.00)	298 (13.84)	
Marital Status (%)				< 0.001
Never married	5948 (17.61)	5359 (17.35)	589 (20.76)	
Married	17350 (55.70)	16306 (57.17)	1044 (37.75)	
Widowed Divorced	2681 (5.83) 3679 (10.35)	2395 (5.66) 3166 (9.77)	286 (7.93) 513 (17.47)	
Separated	1118 (2.35)	920 (2.10)	198 (5.41)	
Living with partner	2713 (8.16)	2429 (7.95)	284 (10.68)	
PIR (%)				< 0.001
Middle Income	16445 (49.34)	15078 (49.11)	1367 (52.17)	
Low Income	6311 (13.66)	5313 (12.38)	998 (29.25)	
High Income	7927 (37.00)	7646 (38.51)	281 (18.58)	
BMI (%)				< 0.001
Healthy weight	8801 (27.73)	8171 (28.02)	630 (24.16)	
Underweight	500 (1.51)	449 (1.47)	51 (1.99)	
Overweight	11004 (32.83)	10250 (33.41)	754 (25.63)	
Obese	12868 (37.94)	11432 (37.10)	1436 (48.22)	
Sleep Duration (%)				< 0.001
Average	20101 (63.72)	18858 (65.17)	1243 (46.04)	
Short	11595 (31.83)	10187 (30.66)	1408 (46.11)	
Long	1722 (4.45)	1479 (4.17)	243 (7.85)	
Substance Use (%)				< 0.001
No	9905 (39.44)	9258 (40.44)	647 (27.85)	
Yes	12308 (60.56)	10906 (59.56)	1402 (72.15)	
Diabetes (%)				< 0.001
No	29072 (90.23)	26759 (90.73)	2313 (84.04)	
Yes	4415 (9.77)	3815 (9.27)	600 (15.96)	
Hypertension (%)				< 0.001
No	23579 (73.79)	21878 (74.81)	1701 (61.32)	
Yes	9830 (26.21)	8621 (25.19)	1209 (38.68)	
Heart Failure (%)				< 0.001
No	32328 (97.60)	29631 (97.88)	2697 (94.17)	
Yes	1097 (2.40)	892 (2.12)	205 (5.83)	
Angina (%)				< 0.001
No	32535 (97.74)	29813 (97.98)	2722 (94.77)	
Yes	876 (2.26)	704 (2.02)	172 (5.23)	
Stroke (%)				< 0.001
No	32195 (97.16)	29524 (97.49)	2671 (93.15)	
Yes	1269 (2.84)	1034 (2.51)	235 (6.85)	
Cancer (%)				0.078
No	30237 (89.67)	27659 (89.79)	2578 (88.14)	
Yes	3247 (10.33)	2911 (10.21)	336 (11.86)	

Abbreviations: PHQ-9 =  nine-item Patient Health Questionnaire; PIR =  ratio of family income to poverty; BMI =  body mass index; SD =  standard deviation.

Categorical characteristics reported as unweighted frequency and weighted percent.

Continuous characteristics reported as weighted mean and standard deviation.

*P*-values <  0.05 denote significant differences between depressive symptoms risk.

With respect to the past 5 days, cigarettes had the highest total usage (6827), followed by cigars (584), chewing tobacco (312), snuff (177), and pipes (46) (S1 Table).

### 3.2. Association between tobacco use and depressive symptoms

Compared to non-tobacco users, individuals with recent cigarette use had higher PHQ-9 scores (adjusted coefficient [aCoef.] =  0.83; 95% Confidence Interval [CI] =  [0.63, 1.04]), higher odds of having depressive symptoms (adjusted odds ratio [aOR] =  1.73; 95% CI =  [1.48, 2.02]) (Table 2), with a gradient of increased odds across different levels of symptom severity compared to minimal symptoms: 1.32 (95% CI =  [1.19, 1.47]) for mild, 1.94 (95% CI =  [1.61, 2.33]) for moderate, 1.71 (95% CI =  [1.32, 2.21]) for moderately severe, and 1.81 (95% CI =  [1.13, 2.89]) for severe symptoms (S2 Table ). In contrast, the use of smoked and smokeless tobacco products was not associated with depressive symptoms, as indicated by adjusted coefficients close to zero with 95% CIs that include zero, or odds ratios close to one with 95% CIs that include one (Table 2 and S2).

**Table 2:** Main effects models for association between tobacco use and total PHQ-9 scores, depressive symptoms (yes/no).

Total PHQ-9 Score				
Tobacco Use	Coef. Estm. (95% CI)	*p*-value	aCoef. Estm. (95% CI)	*p*-value
Cigarettes	1.67 (1.48,1.85)	< 0.001	0.83 (0.63,1.04)	< 0.001
Smoked Tobacco	-0.01 (-0.32,0.30)	0.939	0.09 (-0.23,0.41)	0.572
Smokeless Tobacco	-0.28 (-0.63,0.08)	0.127	-0.29 (-0.65,0.07)	0.117
Depressive Symptoms – Yes				
Tobacco Use	OR (95% CI)	*p*-value	aOR (95% CI)	*p*-value
Cigarettes	2.79 (2.48,3.13)	<0.001	1.73 (1.48,2.02)	<0.001
Smoked Tobacco	1.17 (0.86,1.58)	0.317	1.27 (0.89,1.80)	0.179
Smokeless Tobacco	0.75 (0.47,1.20)	0.223	0.58 (0.33,1.02)	0.060

Note: Coef. Estm. =  unadjusted coefficient estimate, aCoef. Estm. =  adjusted coefficient estimate, OR =  unadjusted odds ratio, aOR =  adjusted odds ratio, CI =  confidence interval, the reference level for depressive symptoms is “No”, the reference level for tobacco use is “Non-Tobacco Use”, p-values <  0.05 denote statistical significance.

**Fig 3:**
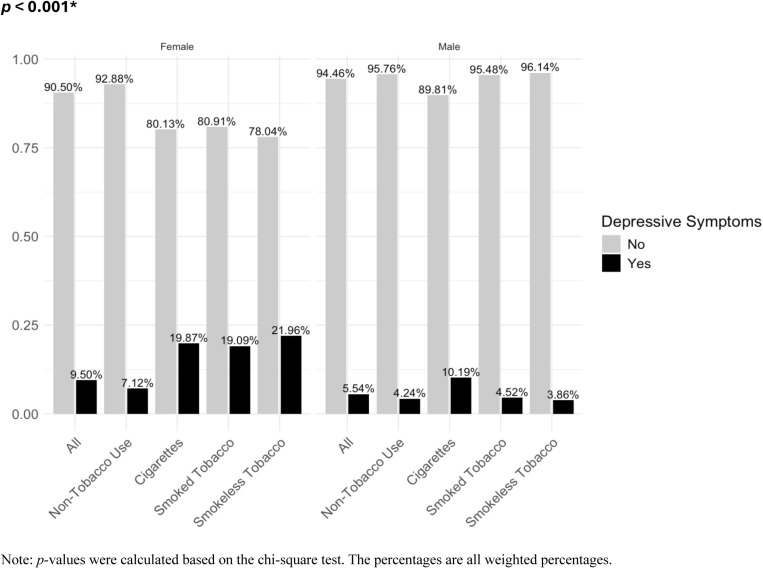
Prevalence of depressive symptoms by tobacco use category stratified by sex.

The interaction models indicated that the relationship between tobacco use and depressive symptoms varied by sex (Table 3, Fig 3, Table 3S). Subgroup analyses (Table 4) demonstrated that for females, cigarette use was associated with 1.23 units increase in total PHQ-9 score (95% CI =  [0.92, 1.55]), indicating a stronger association compared to males (aCoef. =  0.45; 95% CI =  [0.21, 0.69]). Further subgroup analysis showed a positive association between cigarette use and both the presence of depressive symptoms (Table 4) and all severity levels of depressive symptoms in females (S4 Table). However, no association was found in males with moderately severe and severe symptoms (S4 Table).

**Table 3:** Models for interaction of sex and tobacco use on total PHQ-9 scores and depressive symptoms (yes/no).

Total PHQ-9 Score				
Tobacco Use x Sex	Coef. Estm. (95% CI)	*p*-value	aCoef. Estm. (95% CI)	*p*-value
Cigarettes x Female	0.97 (0.67,1.27)	< 0.001	0.90 (0.56,1.24)	< 0.001
Smoked Tobacco x Female	2.36 (1.35,3.38)	< 0.001	1.77 (0.77,2.76)	0.001
Smokeless Tobacco x Female	3.18 (-0.73,7.09)	0.109	4.07 (-2.48,10.62)	0.220
Depressive Symptoms - Yes				
Tobacco Use x Sex	OR (95% CI)	*p*-value	aOR (95% CI)	*p*-value
Cigarettes x Female	1.26 (1.00,1.59)	<0.001	1.29 (0.96,1.74)	0.090
Smoked Tobacco x Female	2.88 (1.55,5.33)	<0.001	2.44 (1.19,5.01)	<0.001
Smokeless Tobacco x Female	4.05 (0.98,16.77)	0.053	5.61 (0.75,41.76)	0.091

Note: Coef. Estm. =  unadjusted coefficient estimate, aCoef. Estm. =  adjusted coefficient estimate, OR =  unadjusted odds ratio, aOR =  adjusted odds ratio, CI =  confidence interval, the reference level for depressive symptoms is “No”, the reference level for sex is “Male”, p-values <  0.05 denote statistical significance.

**Table 4:** Subgroup models following significant interaction between tobacco use and sex.

Total PHQ-9 Score					
Sex		Coef. Estm. (95% CI)	*p*-value	aCoef. Estm. (95% CI)	*p*-value
Female	Cigarettes	2.25 (1.99,2.52)	<0.001	1.23 (0.92,1.55)	<0.001
	Smoked Tobacco	2.32 (1.33,3.30)	<0.001	1.41 (0.46,2.37)	0.004
	Smokeless Tobacco	3.24 (-0.60,7.07)	0.097	3.47 (-2.91,9.85)	0.282
Male	Cigarettes	1.28 (1.07,1.49)	<0.001	0.45 (0.21,0.69)	<0.001
	Smoked Tobacco	-0.04 (-0.34,0.26)	0.778	-0.27 (-0.61,0.06)	0.108
	Smokeless Tobacco	0.06 (-0.31,0.42)	0.766	-0.44 (-0.78,-0.10)	0.011
Depressive Symptoms - Yes					
Sex		OR (95% CI)	*p*-value	aOR (95% CI)	*p*-value
Female	Cigarettes	3.24 (2.81,3.73)	<0.001	1.98 (1.60,2.45)	<0.001
	Smoked Tobacco	3.08 (1.82,5.21)	<0.001	2.18 (1.22,3.89)	0.009
	Smokeless Tobacco	3.67 (1.08,12.44)	0.037	2.58 (0.43,15.53)	0.296
Male	Cigarettes	2.56 (2.11,3.11)	<0.001	1.38 (1.10,1.74)	0.006
	Smoked Tobacco	1.07 (0.74,1.55)	0.721	0.88 (0.56,1.38)	0.560
	Smokeless Tobacco	0.91 (0.52,1.59)	0.729	0.45 (0.24,0.85)	0.014

Note: Coef. Estm. =  unadjusted coefficient estimate, aCoef. Estm. =  adjusted coefficient estimate, OR =  unadjusted odds ratio, aOR =  adjusted odds ratio, CI =  confidence interval, the reference level for depressive symptoms is “No”, the reference level for sex is “Male”, *p*-values <  0.05 denote statistical significance.

Smoked tobacco use was associated with 1.41 units increase in total PHQ-9 scores (95% CI =  [0.46, 2.37]), and 2.18 times higher odds (95% CI =  [1.22, 3.89]) of reporting depressive symptoms in females. Female smoked tobacco users had 3.89 times higher odds (95% CI =  [1.60, 9.45]) of reporting moderately severe symptoms, but due to the small sample size, the wide CI suggests variability in this result. In contrast, male smoked tobacco users had 0.69 times the odds (95% CI =  [0.50, 0.96]) of reporting mild symptoms (S4 Table).

Female smokeless tobacco users did not show a notable association with PHQ-9 scores or presence of depressive symptoms. However, male smokeless tobacco users showed 0.44 units decrease in total PHQ-9 scores (95% CI =  [-0.78, -0.11]) and had 0.45 times the odds (95% CI =  [0.24, 0.85]) of having depressive symptoms (Table 4). Male smokeless tobacco users also had 0.36 times the odds of reporting moderate symptoms (95% CI =  [0.15, 0.83]), while female smokeless tobacco users showed 36.72 times higher odds (95% CI =  [5.67, 237.56]) of having severe symptoms (S4 Table). However, caution is needed for wide CIs due to small sample size, which indicates substantial variability.

### 3.3. Sub-analysis 1: Cognitive-affective and somatic symptoms in cigarettes users vs. non-tobacco users

For all individual symptoms of depression (i.e., distinct items on the PHQ-9), a larger proportion of non-tobacco users reported to not experience depressive symptoms compared to cigarette smokers. Conversely, a higher proportion of cigarette smokers reported experiencing symptoms ‘Several days,’ ‘More than half the days,’ or ‘Nearly every day,’ indicating more frequent depressive symptoms in the cigarette group compared to the non-tobacco use group (S1 Fig).

Participants who used cigarettes had a 0.41-unit increase in cognitive-affective scores (95% CI =  [0.30, 0.51]) and a 0.43-unit increase in somatic scores (95% CI =  [0.31, 0.55])compared to non-tobacco users (S5 Table). Female cigarette smokers had higher total scores in both the cognitive-affective (aCoef. =  0.46; 95% CI =  [0.29, 0.63]) and somatic (aCoef. =  0.43; 95% CI =  [0.23, 0.63]) symptom clusters than males (S6 Table). These findings were further supported by subgroup analysis. Female cigarette users had a 0.60-unit increase in the cognitive-affective symptom cluster (95% CI =  [0.44, 0.77]) and a 0.63-unit increase in the somatic symptom cluster (95% CI =  [0.45, 0.82]). In males, the association was also observed across both symptom clusters but with lower coefficient estimates (aCoef. =  0.21, 95% CI =  [0.09, 0.33] for cognitive-affective, and 0.24, 95% CI =  [0.09, 0.38] for somatic) (S7 Table).

### 3.4. Sub-analysis 2: Detailed examination of cigarette consumption and depressive symptoms

Cigarette consumption, transformed to the logarithm of total cigarettes smoked, was associated with an increase in both total PHQ-9 scores (aCoef. =  0.34; 95% CI =  [0.23, 0.45]) and the presence of depressive symptoms (aOR =  1.33; 95% CI =  [1.22, 1.45]). This trend was consistent across varying degrees of depressive symptom severity, with aORs showing a positive association for both moderate (aOR =  1.37; CI =  [1.21, 1.55]) and moderately severe symptoms (aOR =  1.32; CI =  [1.11, 1.57]) (S8a Table & S8b Table).

## 4. Discussion

In this study, we evaluated the association of depressive symptoms, depressive symptom severity and symptom clusters with tobacco use as well as sex differences in these associations. Individuals who smoked cigarettes exhibited significantly higher odds of depressive symptoms compared to non-tobacco users, with this association being particularly stronger in females than males. Interestingly, the use of other tobacco products, such as smoked and smokeless tobacco, did not show a significant association with depressive symptoms. Further analysis revealed that cigarette consumption, especially among females, was linked to higher scores in both cognitive-affective and somatic symptom clusters, and this pattern remained consistent across different levels of depressive symptom severity. Cigarette use was also significantly linked to the presence of depressive symptoms and increased symptom severity, with the association being strongest for moderate symptoms, though less pronounced for severe symptoms. Additionally, subgroup analysis indicated that female smokers exhibited a stronger connection to depressive symptoms across all severity levels, while males showed a weaker or no association at more severe levels. Smokeless tobacco use in females was linked to severe depressive symptoms, but the small sample size limited the robustness of this finding, suggesting caution in interpretation.

The results of our study are in-line with previous studies that reported the association of cigarette smoking and depression [22,23]. A cross-sectional, community-based study by Stubbs et al. involving 242,952 participants reported a consistent link between smoking behavior and the spectrum of depression across 48 countries [24]. Furthermore, Wu et al. identified a positive association between smoking and depression using NHANES data [25]. However, in this study, the association between depressive symptom severity and symptom clusters was not evaluated. We also identified a positive association between cognitive-affective and somatic symptom cluster scores and smoking, with a stronger association observed for somatic symptoms. The stronger association between smoking and somatic symptoms could be attributed to the direct physiological effects of smoking on the body. Smoking is known to have various harmful effects on physical health, such as inflammation and oxidative stress, which can contribute to the development of somatic symptoms [26]. Additionally, individuals who smoke may also experience heightened awareness of bodily sensations, leading to a greater focus on somatic symptoms [27]. It is possible that individuals experiencing higher somatic symptoms, such as fatigue or pain, may turn to smoking as a form of self-medication or as a coping mechanism [27,28]. Some people perceive smoking as a way to alleviate stress or manage discomfort, even though smoking itself can exacerbate certain symptoms in the long term [28]. This could create a feedback loop where individuals with higher somatic symptoms are more likely to smoke, leading to a stronger association between smoking and somatic symptom clusters.

Several hypotheses can explain the association between cigarette smoking and depressive symptoms [29]. The increased odds of depressive symptoms among smokers might arise if individuals experiencing depressive symptoms use tobacco as a means of self-medication to improve their mental state—the self-medication hypothesis [28]. An alternative perspective suggests that smoking may contribute to depression by impacting an individual's neurocircuitry. Tobacco contains substances, such as nicotine, primarily used to stimulate the brain. Nicotine primarily acts on nicotinic acetylcholine (nACh) receptors, triggering the release of various substances including norepinephrine, serotonin, dopamine, acetylcholine, γ-aminobutyric acid, and glutamate in various brain regions such as the lateral septum, dorsal raphe nucleus, mesolimbic dopamine system, and hippocampus [30]. These brain regions regulate pathways related to stress response, anxiety and depression, thereby impacting anxiety levels and mood [31,32]. Prolonged nicotine exposure leads to to dysregulation of hypothalamic-pituitary-adrenal system, resulting in hypersecretion of cortisol and alterations in the activity of the associated monoamine neurotransmitter system [33]. This can heighten sensitivity and vulnerability, increasing the risk of depressive symptoms. Another key hypothesis suggests that the association between smoking and depressive symptoms may be driven by shared genetic and environmental risk factors. For instance, individuals with a genetic predisposition or those exposed to certain environmental stressors may be more likely to smoke and develop depression, independently of one another. This shared vulnerability model has garnered increasing attention in recent years, as it highlights the complexity of the relationship between smoking and mental health [29].

The observed sex difference in the association between smoking and depression in our study is consistent with previous research [34]. The stronger association between smoking and depression in females observed in our study may be influenced by various factors [35]. Biologically, females may metabolize nicotine differently, leading to a stronger physiological response to smoking, which could increase the odds of developing depressive symptoms [36]. Hormonal fluctuations throughout the menstrual cycle and during menopause could also interact with smoking in a way that impacts mood regulation more significantly in females [37]. Psychosocial factors, such as unique stressors or societal pressures faced by females, could contribute to both smoking initiation and the development of depression [27]. Additionally, females may be more likely to use smoking as a coping mechanism for stress or negative emotions, creating a stronger link between smoking behavior and depressive symptoms [36]. Cultural factors, including norms surrounding emotional expression and coping mechanisms, may also contribute to the observed association [36,37]. As aforementioned, in our study, female cigarette smokers exhibited greater cognitive-affective symptoms compared to males. A notable effect on the somatic symptom cluster was also observed in females. Women may be more likely to use smoking as a coping mechanism for stress and negative emotions, potentially exacerbating cognitive-affective symptoms, especially considering societal pressures and gender roles that could make them more susceptible to the cognitive-affective impact of smoking [36]. Additionally, hormonal fluctuations could also influence the experience of somatic symptoms, making them more pronounced in female smokers [38]. These factors collectively suggest a complex interplay between smoking, sex, hormonal factors, and stress in the manifestation of different symptom clusters in females.

The insignificant association between other tobacco products (i.e., pipes, cigars, chewing tobacco and snuff) and depressive symptoms in our study could be attributed to several factors. Firstly, these alternative tobacco products may deliver nicotine differently than cigarettes, potentially resulting in weaker associations with depressive symptoms. Additionally, individuals who use these products might do so less frequently or in different social or cultural contexts compared to cigarette smokers, impacting the relationship with depressive symptoms. Furthermore, the reasons for using alternative tobacco products could vary widely, with different motivations and stressors compared to cigarette smoking, leading to a weaker observed association. Moreover, the small sample size of users of alternative tobacco products in our study might have limited the statistical power to detect significant associations. This suggests that further research with larger and more diverse samples focusing specifically on these alternative tobacco products is needed to gain a clearer understanding of their relationship with depressive symptoms.

This study has several limitations. Firstly, the cross-sectional design of the NHANES data restricts the ability to establish causal relationships between tobacco use and depressive symptoms. While longitudinal data could provide a clearer temporal relationship, observational methods still present challenges in drawing definitive causal inferences. Secondly, the reliance on self-reported data for both tobacco use and depressive symptoms introduces the possibility of recall bias and social desirability bias. Participants may underreport tobacco use or depressive symptoms due to stigma or social norms, potentially affecting the accuracy of the results. Additionally, the assessment of tobacco use was limited to a few specific types of tobacco products, such as cigarettes, pipes, cigars, chewing tobacco and snuff. Other forms of tobacco or nicotine consumption, such as electronic cigarettes, were not included in the analysis due to a lack of availability in all cycles of NHANES. This may have led to an incomplete picture of overall tobacco use patterns and their associations with depressive symptoms. Furthermore, the NHANES questionnaire assessed tobacco use over the past 5 days, which may not fully capture the chronic nature of tobacco use and its association with depressive symptoms. This is particularly important, as a 2-week duration is typically required for the clinical diagnosis of depression. Finally, the study's sample size for certain subgroups, such as users of alternative tobacco products, was relatively small, which could affect the generalizability of the findings. Despite these limitations, the study provides valuable insights into the association between tobacco use and depressive symptoms using nationally representative data. This study has several strengths that enhance the reliability and depth of its findings. Firstly, the use of nationally representative data from the NHANES allows for the generalizability of the results to the broader non-institutionalized civilian population. This large and diverse dataset provides a foundation for drawing conclusions about the associations between various forms of tobacco use and depressive symptoms. Secondly, the study's comprehensive assessment of tobacco use is a notable strength. By including various forms of tobacco such as cigarettes, pipes, cigars, chewing tobacco and snuff the analysis offers a thorough examination of the potential impacts of different tobacco products on depressive symptoms. This broad approach enables a nuanced understanding of how different types of tobacco use may relate to depressive symptoms in the population.

In conclusion, our study provides valuable insights into the associations between various forms of tobacco use and depressive symptoms. While we found that cigarette smokers had higher cognitive-affective and somatic depressive symptoms, other tobacco products did not exhibit significant associations with depressive symptoms. These results highlight the need for targeted interventions and public health initiatives to address the impact of cigarette smoking on mental health, particularly concerning cognitive-affective and somatic symptoms of depression. Future research should focus not only on longitudinal studies but also on employing a range of complementary research methods, such as triangulation with experimental and quasi-experimental designs, to better understand the complex relationships between tobacco use and depression [39]. This multi-method approach can inform more effective strategies to promote mental health and reduce the burden of depressive symptoms in the population.

## Supporting information

S1 Fig PHQ-9 symptom profile proportions: non-tobacco use vs cigarette. (DOCX)

S1 Table Quantitative overview of tobacco product usage. (DOCX)

S2 Table Main effects models for association between tobacco use and depressive symptom severity. (DOCX)

S3 Table Models for interaction of sex and tobacco use on depressive symptom severity. (DOCX)

S4 Table Subgroup models following significant interaction between tobacco use and sex. (DOCX)

S5 Table Main effects models for association between tobacco use (cigarettes vs non-tobacco use) and symptom clusters. (DOCX)

S6 Table Models for interaction of sex and tobacco use (cigarettes vs non-tobacco use) on symptom clusters. (DOCX)

S7 Table Subgroup models following significant interaction between tobacco use (cigarettes vs non-tobacco use) and sex. (DOCX)

S8a Table Main effects models for association between total cigarettes and total PHQ-9 scores, depressive symptoms (yes/no). (DOCX)

S8b Table Main effects models for association between total cigarettes and depressive symptom severity. (DOCX)
