# Supplementary figures and images for: Association of tobacco use with depressive symptoms in adults: Considerations of symptom severity, symptom clusters, and sex
Source: PLoS One. 2025 Apr 2;20(4):e0319070. doi: 10.1371/journal.pone.0319070 (PMC11964252; doi:10.1371/journal.pone.0319070)

**Figure S1**. PHQ-9 Symptom Profile Proportions: Non-Tobacco Use vs Cigarettes

**
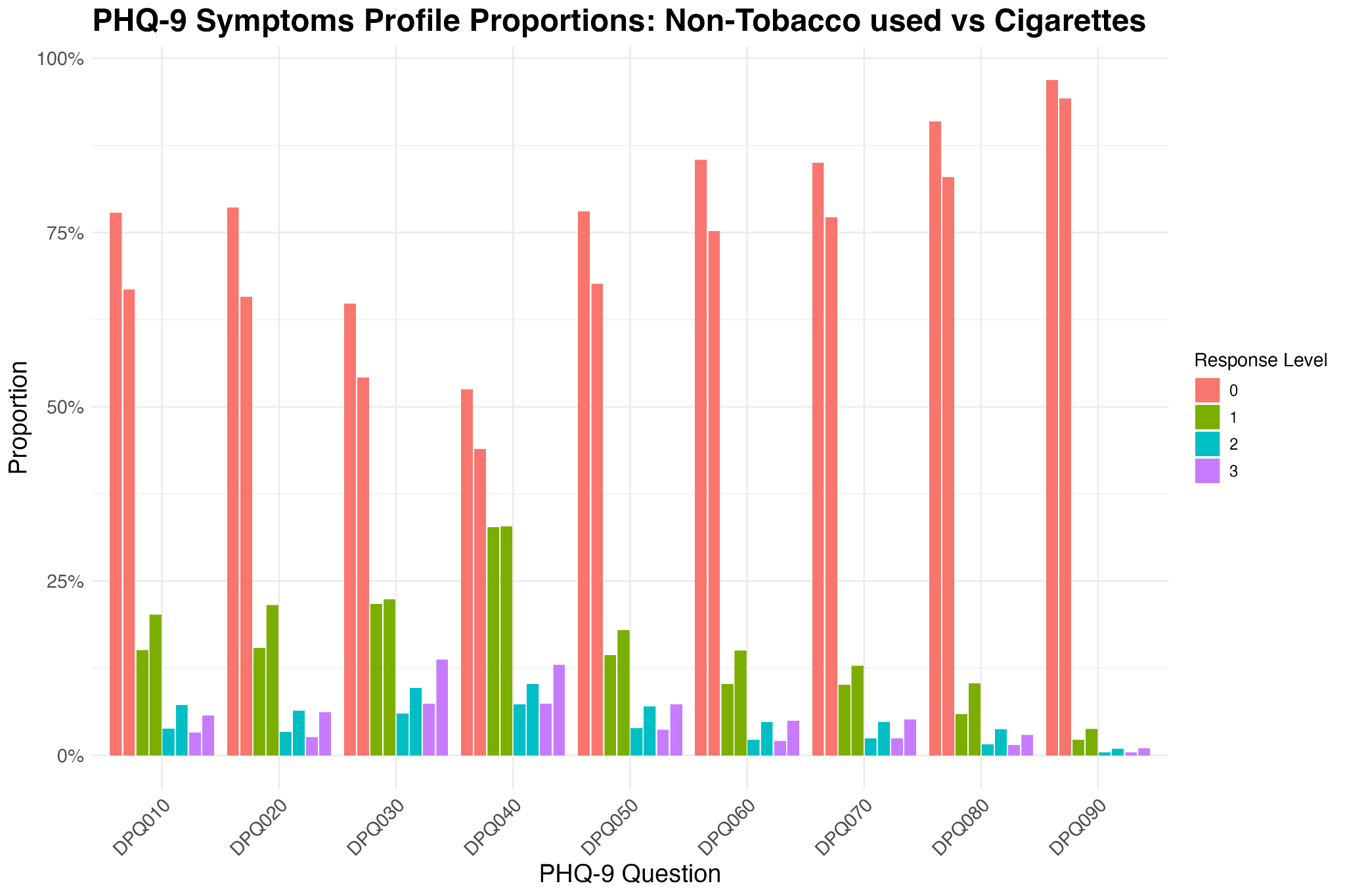
**

Supplement: S1 Fig — (DOCX) [file pone.0319070.s001.docx]
