# Supplementary material for: Association of tobacco use with depressive symptoms in adults: Considerations of symptom severity, symptom clusters, and sex
Source: PLoS One. 2025 Apr 2;20(4):e0319070. doi: 10.1371/journal.pone.0319070 (PMC11964252; doi:10.1371/journal.pone.0319070)
Supplement: S1 Table — (DOCX) [file pone.0319070.s002.docx]

**Table S1.** Quantitative Overview of Tobacco Product Usage

| **# days used over last 5 days** | **Cigarettes** | **Pipes** | **Cigars** | **Chewing Tobacco** | **Snuff** |
| --- | --- | --- | --- | --- | --- |
| **1** | 379 | 10 | 153 | 25 | 7 |
| **2** | 336 | 3 | 67 | 13 | 7 |
| **3** | 333 | 7 | 52 | 13 | 4 |
| **4** | 677 | 11 | 85 | 57 | 30 |
| **5** | 5102 | 15 | 227 | 204 | 129 |
| **SUM** | 6827 | 46 | 584 | 312 | 177 |

Note: The data is based on the unweighted response from SMQ710 (Cigarettes), SMQ740 (Pipes), SMQ770 (Cigars), SMQ800 (Chewing Tobacco), and SMQ817 (Snuff).
