# Supplementary material for: Association of tobacco use with depressive symptoms in adults: Considerations of symptom severity, symptom clusters, and sex
Source: PLoS One. 2025 Apr 2;20(4):e0319070. doi: 10.1371/journal.pone.0319070 (PMC11964252; doi:10.1371/journal.pone.0319070)
Supplement: S2 Table — (DOCX) [file pone.0319070.s003.docx]

**Table S2.** Main effects models for association between tobacco use and depressive symptom severity

| **Tobacco Use** | **Depressive Symptom Severity** | | | | | | | |
| --- | --- | --- | --- | --- | --- | --- | --- | --- |
|  | Mild | | Moderate | | Moderately Severe | | Severe | |
|  | OR  (95% CI) | *p*-value | OR  (95% CI) | *p*-value | OR  (95% CI) | *p*-value | OR  (95% CI) | *p*-value |
| Cigarettes | 1.80  (1.66,1.94) | **<0.001** | 2.99  (2.59,3.45) | **<0.001** | 3.18  (2.61,3.88) | **<0.001** | 3.83  (2.80,5.23) | **<0.001** |
| Smoked Tobacco | 0.83  (0.66,1.03) | 0.097 | 1.03  (0.72,1.48) | 0.872 | 1.44  (0.86,2.39) | 0.164 | 1.06  (0.46,2.44) | 0.900 |
| Smokeless Tobacco | 0.91  (0.65,1.28) | 0.592 | 0.69  (0.36,1.33) | 0.267 | 0.59  (0.23,1.49) | 0.267 | 1.47  (0.53,4.06) | 0.455 |
|  | aOR  (95% CI) | *p*-value | aOR  (95% CI) | *p*-value | aOR  (95% CI) | *p*-value | aOR  (95% CI) | *p*-value |
| Cigarettes | 1.32  (1.19,1.47) | **<0.001** | 1.94  (1.61,2.33) | **<0.001** | 1.71  (1.32,2.21) | **<0.001** | 1.81  (1.13,2.89) | **0.014** |
| Smoked Tobacco | 0.84  (0.65,1.08) | 0.174 | 1.09  (0.70,1.70) | 0.696 | 1.74  (0.99,3.04) | 0.052 | 0.73  (0.22,2.42) | 0.604 |
| Smokeless Tobacco | 1.00  (0.68,1.47) | 0.999 | 0.37  (0.17,0.84) | **0.016** | 0.61  (0.20,1.85) | 0.386 | 1.78  (0.57,5.63) | 0.324 |

Note: OR = unadjusted odds ratio, aOR = adjusted odds ratio, CI = confidence interval, ref = reference level, the reference level for depressive symptoms severity is “Minimal”, the reference level for tobacco use is “Non-Tobacco Use”, *p*-values < 0.05 denote statistical significance.
