# Supplementary material for: Association of tobacco use with depressive symptoms in adults: Considerations of symptom severity, symptom clusters, and sex
Source: PLoS One. 2025 Apr 2;20(4):e0319070. doi: 10.1371/journal.pone.0319070 (PMC11964252; doi:10.1371/journal.pone.0319070)
Supplement: S3 Table — (DOCX) [file pone.0319070.s004.docx]

**Table S3.** Models for interaction of sex and tobacco use on depressive symptom severity.

| **Tobacco Use x Sex** | **Depressive Symptom Severity** | | | | | | | |
| --- | --- | --- | --- | --- | --- | --- | --- | --- |
|  | Mild | | Moderate | | Moderately Severe | | Severe | |
|  | OR  (95% CI) | *p*-value | OR  (95% CI) | *p*-value | OR  (95% CI) | *p*-value | OR  (95% CI) | *p*-value |
| Cigarettes x Female | 1.03  (0.86,1.23) | 0.764 | 1.06  (0.78,1.43) | 0.710 | 1.55  (1.02,2.35) | **0.041** | 3.80  (2.34,6.16) | **<0.001** |
| Smoked Tobacco x Female | 2.15  (1.19,3.87) | **0.011** | 2.40  (0.97,5.93) | 0.057 | 5.57  (1.99,15.59) | **0.001** | 4.69  (0.76,28.86) | 0.095 |
| Smokeless Tobacco x Female | 1.07  (0.37,3.08) | 0.907 | 1.69  (0.44,6.46) | 0.443 | 1.56  (0.16,14.82) | 0.698 | 40.28  (4.40,368.35) | **0.001** |
|  | aOR  (95% CI) | *p*-value | aOR  (95% CI) | *p*-value | aOR  (95% CI) | *p*-value | aOR  (95% CI) | *p*-value |
| Cigarettes x Female | 1.03  (0.83,1.27) | 0.812 | 1.05  (0.72,1.55) | 0.799 | 1.89  (1.16,3.07) | **0.011** | 3.51  (1.94,6.33) | **<0.001** |
| Smoked Tobacco x Female | 1.85  (1.03,3.34) | **0.040** | 2.40  (0.90,6.42) | 0.081 | 4.25  (1.26,14.33) | **0.020** | 0.00  (0.00,0.00) | **<0.001*** |
| Smokeless Tobacco x Female | 1.00  (0.17,5.95) | 1.000 | 0.00  (0.00,0.00) | **<0.001*** | 0.00  (0.00,0.00) | **<0.001*** | 63.21  (6.58,606.94) | **<0.001** |

Note: OR = unadjusted odds ratio, aOR = adjusted odds ratio, CI = confidence interval, ref = reference level, the reference level for depressive symptoms severity is “Minimal”, the reference level for tobacco use is “Non-Tobacco Use”, *p*-values < 0.05 denote statistical significance.

*: While the p-values indicate statistical significance, the aORs and their CIs are exceedingly small, making the results unreliable.
