# Supplementary material for: Association of tobacco use with depressive symptoms in adults: Considerations of symptom severity, symptom clusters, and sex
Source: PLoS One. 2025 Apr 2;20(4):e0319070. doi: 10.1371/journal.pone.0319070 (PMC11964252; doi:10.1371/journal.pone.0319070)
Supplement: S4 Table — (DOCX) [file pone.0319070.s005.docx]

**Table S4.** Subgroup models following significant interaction between tobacco use and sex.

|  | **Tobacco Use** | **Depressive Symptom Severity** | | | | | | | |
| --- | --- | --- | --- | --- | --- | --- | --- | --- | --- |
|  |  | Mild | | Moderate | | Moderately Severe | | Severe | |
|  |  | OR  (95% CI) | *p*-value | OR  (95% CI) | *p*-value | OR  (95% CI) | *p*-value | OR  (95% CI) | *p*-value |
| Female | Cigarettes | 1.92  (1.71,  2.16) | **<0.001** | 3.32  (2.78,  3.98) | **<0.001** | 4.04  (3.13,  5.20) | **<0.001** | 6.61  (4.66,  9.38) | **<0.001** |
|  | Smoked Tobacco | 1.84  (1.18,  2.87) | **0.007** | 2.69  (1.27,  5.72) | **0.010** | 5.71  (2.79,  11.72) | **<0.001** | 3.60  (0.94,  13.76) | 0.061 |
|  | Smokeless Tobacco | 1.25  (0.47,  3.35) | 0.660 | 1.71  (0.47,  6.23) | 0.413 | 1.24  (0.16,  9.71) | 0.840 | 31.15  (5.10,  190.12) | **<0.001** |
| Male | Cigarettes | 1.87  (1.65,  2.11) | **<0.001** | 3.14  (2.46,  4.00) | **<0.001** | 2.61  (1.88,  3.62) | **<0.001** | 1.74  (1.09,  2.77) | **0.020** |
|  | Smoked Tobacco | 0.86  (0.64,  1.14) | 0.285 | 1.12  (0.72,  1.75) | 0.616 | 1.03  (0.48,  2.21) | 0.947 | 0.77  (0.25,  2.37) | 0.645 |
|  | Smokeless Tobacco | 1.17  (0.83,  1.66) | 0.375 | 1.01  (0.51,  2.03) | 0.969 | 0.79  (0.29,  2.13) | 0.644 | 0.77  (0.23,  2.64) | 0.681 |
|  | | aOR  (95% CI) | *p*-value | aOR  (95% CI) | *p*-value | aOR  (95% CI) | *p*-value | aOR  (95% CI) | *p*-value |
| Female | Cigarettes | 1.38  (1.19,  1.60) | **<0.001** | 2.07  (1.59,  2.68) | **<0.001** | 2.14  (1.58,  2.89) | **<0.001** | 3.35  (1.88,  5.98) | **<0.001** |
|  | Smoked Tobacco | 1.40  (0.86,  2.30) | 0.178 | 2.01  (0.92,  4.41) | 0.080 | 3.89  (1.60,  9.45) | **0.003** | 0.00  (0.00, 0.00) | **<0.001*** |
|  | Smokeless Tobacco | 1.03  (0.20,  5.33) | 0.969 | 0.00  (0.00, 0.00) | **<0.001*** | 0.00  (0.00, 0.00) | **<0.001*** | 36.72  (5.67,  237.56) | **<0.001** |
| Male | Cigarettes | 1.25  (1.05,  1.49) | **0.012** | 1.78  (1.35,  2.35) | **<0.001** | 1.13  (0.72,  1.77) | 0.593 | 0.69  (0.35,  1.33) | 0.266 |
|  | Smoked Tobacco | 0.69  (0.50,  0.96) | **0.029** | 0.80  (0.46,  1.41) | 0.449 | 0.94  (0.40,  2.23) | 0.892 | 0.66  (0.19,  2.27) | 0.508 |
|  | Smokeless Tobacco | 0.98  (0.66,  1.46) | 0.922 | 0.36  (0.15,  0.83) | **0.017** | 0.60  (0.19,  1.82) | 0.364 | 0.54  (0.17,  1.75) | 0.308 |

Note: OR = unadjusted odds ratio, aOR = adjusted odds ratio, CI = confidence interval, ref = reference level, the reference level for depressive symptoms severity is “Minimal”, the reference level for tobacco use is “Non-Tobacco Use”, *p*-values < 0.05 denote statistical significance.

*: While the p-values indicate statistical significance, the aORs and their CIs are exceedingly small, making the results unreliable.
