# Supplementary material for: Association of tobacco use with depressive symptoms in adults: Considerations of symptom severity, symptom clusters, and sex
Source: PLoS One. 2025 Apr 2;20(4):e0319070. doi: 10.1371/journal.pone.0319070 (PMC11964252; doi:10.1371/journal.pone.0319070)
Supplement: S5 Table — (DOCX) [file pone.0319070.s006.docx]

**Table S5.** Main effects models for association between tobacco use (cigarettes vs non-tobacco use) and symptom clusters

| **Cognitive-Affective Symptom Cluster** | | | | |
| --- | --- | --- | --- | --- |
| **Tobacco Use** | Coef. Estm.  (95% CI) | *p*-value | aCoef. Estm.  (95% CI) | *p*-value |
| Cigarettes | 0.82  (0.73,0.92) | **<0.001** | 0.41  (0.30,0.51) | **<0.001** |
| **Somatic Symptom Cluster** | | | | |
| **Tobacco Use** | Coef. Estm.  (95% CI) | *p*-value | aCoef. Estm.  (95% CI) | *p*-value |
| Cigarettes | 0.84  (0.74,0.95) | **<0.001** | 0.43  (0.31,0.55) | **<0.001** |

Note: Coef. Estm. = unadjusted coefficient estimate, aCoef. Estm. = adjusted coefficient estimate, CI = confidence interval, ref = reference level, the reference level for tobacco use is “Non-Tobacco Use”, *p*-values < 0.05 denote statistical significance.
