# Supplementary material for: Association of tobacco use with depressive symptoms in adults: Considerations of symptom severity, symptom clusters, and sex
Source: PLoS One. 2025 Apr 2;20(4):e0319070. doi: 10.1371/journal.pone.0319070 (PMC11964252; doi:10.1371/journal.pone.0319070)
Supplement: S6 Table — (DOCX) [file pone.0319070.s007.docx]

**Table S6.** Models for interaction of sex and tobacco use (cigarettes vs non-tobacco use) on symptom clusters

| **Cognitive-Affective Symptom Cluster** | | | | |
| --- | --- | --- | --- | --- |
| **Tobacco Use x Sex** | Coef. Estm.  (95% CI) | *p*-value | aCoef. Estm.  (95% CI) | *p*-value |
| Cigarettes x Female | 0.47  (0.31,0.63) | **<0.001** | 0.46  (0.29,0.63) | **<0.001** |
| **Somatic Symptom Cluster** | | | | |
| **Tobacco Use x Sex** | Coef. Estm.  (95% CI) | *p*-value | aCoef. Estm.  (95% CI) | *p*-value |
| Cigarettes x Female | 0.50  (0.33,0.68) | **<0.001** | 0.43  (0.23,0.63) | **<0.001** |

Note: Coef. Estm. = unadjusted coefficient estimate, aCoef. Estm. = adjusted coefficient estimate, CI = confidence interval, ref = reference level, the reference level for tobacco use is “Non-Tobacco Use”, the reference level for sex is “Male”, *p*-values < 0.05 denote statistical significance.
