# Supplementary material for: Association of tobacco use with depressive symptoms in adults: Considerations of symptom severity, symptom clusters, and sex
Source: PLoS One. 2025 Apr 2;20(4):e0319070. doi: 10.1371/journal.pone.0319070 (PMC11964252; doi:10.1371/journal.pone.0319070)
Supplement: S7 Table — (DOCX) [file pone.0319070.s008.docx]

**Table S7.** Subgroup models following significant interaction between tobacco use (cigarettes vs non-tobacco use) and sex

| **Cognitive-Affective Symptom Cluster** | | | | | |
| --- | --- | --- | --- | --- | --- |
| **Sex** | **Tobacco Use** | Coef. Estm.  (95% CI) | *p*-value | aCoef. Estm.  (95% CI) | *p*-value |
| Female | Cigarettes | 1.10  (0.96,1.23) | **<0.001** | 0.60  (0.44,0.77) | **<0.001** |
| Male | Cigarettes | 0.63  (0.52,0.74) | **<0.001** | 0.21  (0.09,0.33) | **0.001** |
| **Somatic Symptom Cluster** | | | | | |
| **Sex** | **Tobacco Use** | Coef. Estm.  (95% CI) | *p*-value | aCoef. Estm.  (95% CI) | *p*-value |
| Female | Cigarettes | 1.16  (1.00,1.31) | **<0.001** | 0.63  (0.45,0.82) | **<0.001** |
| Male | Cigarettes | 0.65  (0.54,0.77) | **<0.001** | 0.24  (0.09,0.38) | **0.002** |

Note: Coef. Estm. = unadjusted coefficient estimate, aCoef. Estm. = adjusted coefficient estimate, CI = confidence interval, ref = reference level, the reference level for tobacco use is “Non-Tobacco Use”, *p*-values < 0.05 denote statistical significance.
