# Supplementary material for: Association of tobacco use with depressive symptoms in adults: Considerations of symptom severity, symptom clusters, and sex
Source: PLoS One. 2025 Apr 2;20(4):e0319070. doi: 10.1371/journal.pone.0319070 (PMC11964252; doi:10.1371/journal.pone.0319070)
Supplement: S8a Table — (DOCX) [file pone.0319070.s009.docx]

**Table S8a.** Main effects models for association between total cigarettes and total PHQ-9 scores, depressive symptoms (yes/no).

| **Total PHQ-9 Score** | | | | |
| --- | --- | --- | --- | --- |
|  | Coef. Estm.  (95% CI) | *p*-value | aCoef. Estm.  (95% CI) | *p*-value |
| Log (Total Cigarettes) | 0.37  (0.27,0.47) | **<0.001** | 0.34  (0.23,0.45) | **<0.001** |
| **Depressive Symptoms - Yes** | | | | |
|  | OR  (95% CI) | *p*-value | aOR  (95% CI) | *p*-value |
| Log (Total Cigarettes) | 1.28  (1.18,1.37) | **<0.001** | 1.33  (1.22,1.45) | **<0.001** |
