# Supplementary material for: Association of tobacco use with depressive symptoms in adults: Considerations of symptom severity, symptom clusters, and sex
Source: PLoS One. 2025 Apr 2;20(4):e0319070. doi: 10.1371/journal.pone.0319070 (PMC11964252; doi:10.1371/journal.pone.0319070)
Supplement: S8b Table — (DOCX) [file pone.0319070.s010.docx]

**Table S8b.** Main effects models for association between total cigarettes and depressive symptom severity.

|  | **Depressive Symptom Severity** | | | | | | | |
| --- | --- | --- | --- | --- | --- | --- | --- | --- |
|  | Mild | | Moderate | | Moderately Severe | | Severe | |
|  | OR  (95% CI) | *p*-value | OR  (95% CI) | *p*-value | OR  (95% CI) | *p*-value | OR  (95% CI) | *p*-value |
| Log (Total Cigarettes) | 1.05  (0.99,1.12) | 0.113 | 1.28  (1.17,1.41) | **<0.001** | 1.31  (1.13,1.52) | **<0.001** | 1.31  (1.10,1.54) | **0.002** |
|  | aOR  (95% CI) | *p*-value | aOR  (95% CI) | *p*-value | aOR  (95% CI) | *p*-value | aOR  (95% CI) | *p*-value |
| Log (Total Cigarettes) | 1.05  (0.96,1.14) | 0.290 | 1.37  (1.21,1.55) | **<0.001** | 1.32  (1.11,1.57) | **0.002** | 1.29  (0.94,1.76) | 0.116 |

Note: Coef. Estm. = unadjusted coefficient estimate, aCoef. Estm. = adjusted coefficient estimate, OR = unadjusted odds ratio, aOR = adjusted odds ratio, CI = confidence interval, ref = reference level, the reference level for depressive symptoms is “No”, the reference level for depressive symptoms severity is “Minimal”, *p*-values < 0.05 denote statistical significance.
